# Two-stage interrupted time series analysis with machine learning: evaluating the health effects of the 2018 wildfire smoke event in San Francisco County as a case study

**DOI:** 10.1093/aje/kwaf147

**Published:** 2025-07-11

**Authors:** Arnab K Dey, Yiqun Ma, Gabriel Carrasco-Escobar, Changwoo Han, François Rerolle, Tarik Benmarhnia

**Affiliations:** Scripps Institution of Oceanography, University of California, San Diego, CA, United States; Herbert Wertheim School of Public Health and Human Longevity Science, University of California, San Diego, CA, United States; Scripps Institution of Oceanography, University of California, San Diego, CA, United States; Health Innovation Laboratory, Institute of Tropical Medicine ‘Alexander von Humboldt’, Universidad Peruana Cayetano Heredia, Lima, Peru; Scripps Institution of Oceanography, University of California, San Diego, CA, United States; Department of Preventive Medicine, Chungnam National University College of Medicine, Daejeon, South Korea; Scripps Institution of Oceanography, University of California, San Diego, CA, United States; Francis I. Proctor Foundation, University of California, San Francisco, CA, United States; Scripps Institution of Oceanography, University of California, San Diego, CA, United States; Irset Institut de Recherche en Santé, Environnement et Travail, UMR-S 1085, Inserm, University of Rennes, EHESP, Rennes, France

**Keywords:** causal inference, interrupted time series analysis, wildfire smoke, respiratory hospitalization, California

## Abstract

Randomized controlled trials (RCTs) are considered a key identification strategy for establishing causal relationships between exposures and outcomes. When evaluating the health impacts of extreme weather events, however, RCTs are generally infeasible due to ethical issues, costs, and the lack of a suitable control group. Quasi-experimental designs capitalizing on the timing of natural experiments, such as Interrupted Time Series (ITS), offer a valuable alternative to estimate causal effects when control groups are not available. This paper explores the application of a 2-stage ITS framework that compares traditional autoregressive integrated moving average (ARIMA) models and 2 machine learning algorithms: Neural Network Autoregressive (NNETAR) and Prophet-Extreme Gradient Boosting (XGBoost). As a case study, we assess the impacts of the 2018 wildfire smoke event on respiratory hospitalizations in San Francisco County, CA. We split the data into pre- and postevent periods to train and evaluate the models, perform cross-validation for hyperparameter tuning, and predict hospitalizations under the counterfactual scenario. Data and R code are provided for reproducibility. In the case study, the Prophet-XGBoost shows the best model performance and was used to generate the counterfactual trends. We estimate that the 2018 smoke event resulted in a total of 92 (95% empirical CI, 24-125) excess respiratory hospitalizations (12.5% of the observed hospitalization count during the event period). Our proposed approach offers a powerful tool for assessing the effects of extreme weather events and can be broadly applied to other epidemiological contexts, such as public health policy evaluation.

## Introduction

Randomized controlled trials (RCTs) are widely regarded as a key identification strategy to infer causal effects. However, RCTs are not always desirable or feasible due to ethical concerns, costs, or simply because the intervention of interest has already been implemented. This is particularly true when evaluating the health impacts of extreme weather events, which often occur on a large scale and cannot be randomly and ethnically assigned to specific populations. In this context, alternative quasi-experimental designs have been advanced, which capitalize on the timing of natural experiments to mimic randomization, enabling the estimation of causal effects using observational data to evaluate the impacts of extreme weather events or public health interventions.^1^^-^^3^

Most quasi-experimental methods compare an outcome of interest in a population exposed to an event, such as an extreme weather event, with a control group that is not exposed to the event.^2^^,^^3^ However, control groups are not always available. This can be due to limited data availability or because all available geographical units were exposed at the same time. In this context, there are some approaches that compare the effects of an exposure within a single exposed population.^1^ Such designs are valuable in contexts where interventions are implemented arbitrarily (ie, without randomization) or where exposures occur in a whole population (ie, without a comparison group).^1^^,^^4^ One such design is the Interrupted Time Series (ITS) analysis, which is well suited to evaluate the effects of exposures occurred at a clearly defined time point.^5^

The ITS approach assumes that an event will change either the magnitude or direction of an outcome following its occurrence and leverages the randomness in the timing of the event to detect these changes. This is achieved by collecting time series data on an outcome of interest at multiple time points before and after an exposure event. This approach then extrapolates the time series data from the pre-event phase to generate a counterfactual trend that represents what would have been observed (under specific assumptions) in the absence of the exposure event. The effect of an event is then estimated by comparing the actual time series in the postevent period with the counterfactual time series.^4^ By studying a single population over a period of time, the ITS design can avoid issues related to differences between groups, like selection bias or unmeasured confounders.^4^

Generating counterfactual trends for ITS typically involves methods such as autoregressive integrated moving average (ARIMA),^6^ which assumes that the time trends in the pre- and postevent periods can be modeled as a linear combination of parameters. Such traditional ITS designs have been shown to generate biased estimates when certain underlying assumptions are not met.^7^ In addition, most of the ITS literature (both empirical examples and tutorials) focused on a single-stage model where all data units (pre- and post-data points) are included, and models are optimized based on a stepwise approach (eg, using the Akaike criterion). In parallel, some researchers proposed a 2-stage ITS framework,^8^^,^^9^ where the identification of causal effects is decomposed into 2 distinct steps: (1) using the pre-event data only, optimize a predictive model that will fit the observed data as best as possible based on calendar/seasonal and time-varying variables; and (2) once the predictive model in optimized in step 1, applying it to postevent data to compare with observed outcomes and infer the potential effects related to the exposure over time. A 2-stage approach offers a few advantages over traditional 1-stage ITS approaches. First, it allows for a careful examination of outcome trends in the pre-event period using multiple modeling approaches. This initial stage enables researchers to check whether the models and available data can adequately capture the underlying temporal patterns of the outcome before the event, which is essential for generating a valid counterfactual trend in the postevent period within an ITS design. In cases where pre-event trends cannot be adequately captured, researchers may opt for alternative causal inference methods that utilize different strategies for constructing counterfactuals. Second, this approach does not rely on the parametrization of changes in intercepts and slopes, as treatment effects can be estimated directly and for each time unit after the occurrence of the event, which offers more modeling flexibility.

Many recent machine learning (ML) approaches have demonstrated high performance in predicting trends through data-driven processes. These approaches allow nonlinear interactions between covariates and complex functional forms, and they have been shown to outperform predictions generated by linear approaches.^10^ While some ML approaches have been successfully used in prior studies in conjunction with ITS,^11^^,^^12^ they have not been used or compared in the context of 2-stage ITS analyses. In this paper, we aim to describe how ML algorithms can be used in the context of 2-stage ITS and compare the performance of multiple algorithms. Specifically, we will focus on Neural Network Autoregressive (NNETAR) and Prophet-Extreme Gradient Boosting (XGBoost) hybrid models and compare their performances to the traditional ARIMA model. We demonstrate the use of these methods, estimating the impacts of the 2018 wildfire smoke event on respiratory hospitalizations in San Francisco County, CA, as an illustrative case study.

The 2018 Camp Fire in Butte County, Northern California, which began on November 8, 2018, and burned for more than 2 weeks, was one of the most destructive wildfires in the state’s history. It directly caused 85 fatalities, displaced over 50 000 people, and destroyed more than 18 000 structures, resulting in an estimated $16.5 billion in damage.^13^ Smoke from the Camp Fire traveled south and west into the San Francisco County, driving the county’s 24-hour average fine particulate matter (PM_2.5_) concentrations to exceed 35 μg/m^3^—above the US Environmental Protection Agency (EPA)’s primary standard—for 12 consecutive days, with a peak of 105 μg/m^3^. Wildfire PM_2.5_ has been shown to affect respiratory health.^14^^,^^15^ However, the impact of this 2018 wildfire smoke event on respiratory hospitalizations in San Francisco County has not been quantified yet.

This paper introduces a hybrid methodology that integrates multiple ML algorithms within a 2-stage ITS analysis, aiming at enhancing the robustness of the ITS approach for causal inference. We demonstrated the application of this hybrid approach through a case study that assessed the impacts of the 2018 wildfire smoke event on respiratory hospitalizations in San Francisco County, CA.

## Methods

### Study overview

#### Methodological framework

This study introduced an observational 2-stage ITS design coupled with multiple algorithms to generate counterfactual trends. As a case study, we conducted a longitudinal analysis using daily data on respiratory hospitalization and environmental variables from 2009 to 2018 in San Francisco County, CA.

**Figure 1:**
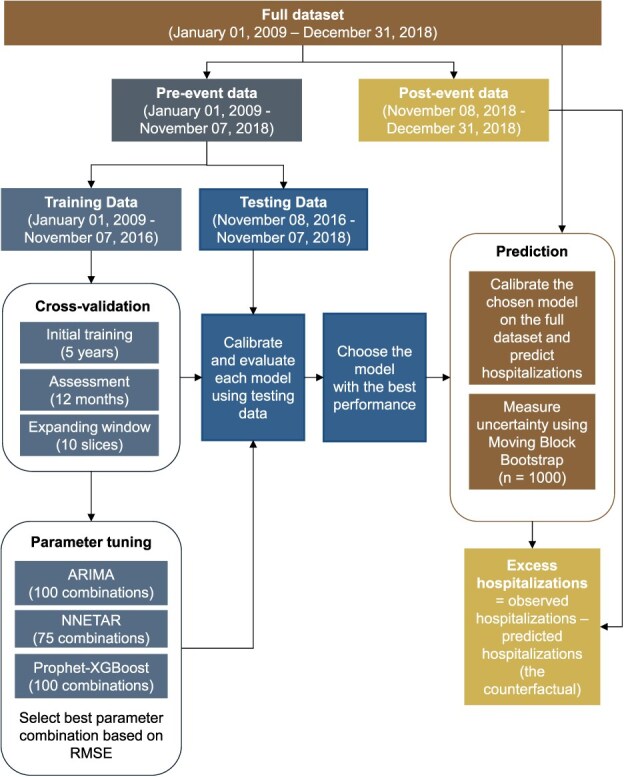
Methodology framework for estimating excess respiratory hospitalizations due to the smoke event in San Francisco County.

First, we used data before the 2018 wildfire smoke event to train and test 3 models: a traditional ARIMA model, a neural network designed for time series autoregression (NNETAR), and a hybrid model that combines Prophet algorithm with XGBoost (Prophet-XGBoost). Then, the model with the best performance was selected as the primary model to predict expected respiratory hospitalizations during and after the smoke event, representing the counterfactual scenario in which the smoke event did not occur. Finally, the difference between the observed and expected respiratory hospitalizations during the event period was calculated to determine the excess hospitalizations attributable to the smoke event. Figure 1 presents an overview of the methodology of the study.

#### Wildfire smoke event period

**Figure 2:**
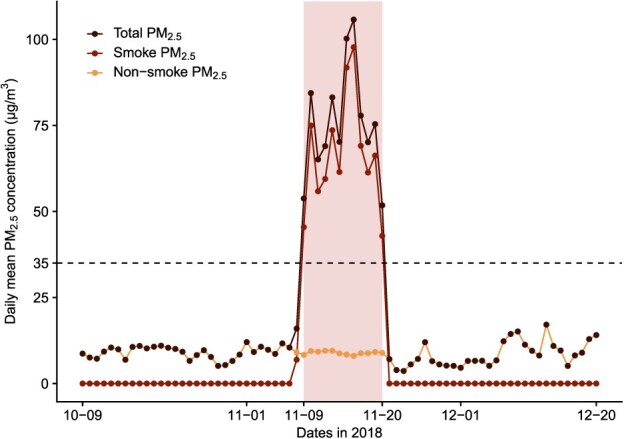
Daily mean PM_2.5_ concentrations in San Francisco County, before, during, and after the 2018 wildfire smoke event. This figure shows the time series of the daily mean total PM_2.5_, wildfire smoke PM_2.5_, and nonsmoke PM_2.5_ concentrations (μg/m^3^) in San Francisco County, before, during, and after the 2018 wildfire smoke event. The horizontal dashed line represents the US Environmental Protection Agency (EPA)‘s 24-hour average standard for PM_2.5_ (35 μg/m^3^). The shaded rectangle marks the period of the smoke event (November 9-20, 2018), when the daily mean total PM_2.5_ concentrations exceeded the EPA’s standard. The lines of total PM_2.5_ and nonsmoke PM_2.5_ overlap on days without smoke PM_2.5_.

In the main analysis, we defined the 2018 smoke event period as the 12 consecutive days (November 9-20, 2018) during which the daily mean PM_2.5_ concentration in San Francisco County exceeded the US EPA’s primary standard of 35 μg/m^3^ (Figure 2). Considering that the Camp Fire began in its source location on November 8, 2018, we selected an alternative event period of November 8-20, 2018, for secondary analysis. Additionally, to account for the potentially delayed health effects of smoke exposure, we estimated the excess hospitalizations in the week following the smoke event as well (November 21-27, 2018).

### Data sources

#### Respiratory hospitalization data

Our study analyzed respiratory hospitalization data from 2 complementary sources maintained by the California Department of Health Care Access and Information, collectively referred to hereafter as unscheduled hospitalizations: the Patient Discharge Data, which captures inpatient admissions, and the Emergency Department Data, which records emergency department visits.^16^ Using both databases enabled us to comprehensively capture the full spectrum of unscheduled respiratory healthcare encounters, including cases treated solely in emergency settings and those requiring inpatient admission. We calculated daily unscheduled respiratory hospitalization counts at the county level by aggregating ZIP code-level data based on patients’ residential addresses. To identify respiratory hospitalizations, we utilized primary diagnosis codes for Diseases of the Respiratory System from the primary International Classification of Diseases (ICD)-9 and ICD-10 coding systems. The specific diagnosis codes are detailed in Appendix S1. This study was approved by the California Health and Human Services Agency’s Committee for the Protection of Human Subjects (project number: 2021-116).

#### Environmental data

Dail ZIP Code Tabulation Area (ZCTA)-level total and wildfire-specific PM_2.5_ concentrations at population centroids were estimated using an ensemble-based statistical approach in a previous study and were averaged to the county level.^17^ Nonsmoke PM_2.5_ concentration was calculated as the difference between total PM_2.5_ and wildfire-specific PM_2.5_. Daily maximum temperature, minimum temperature, dew point temperature, total precipitation, and nonsmoke PM_2.5_ concentration were used as predictors for our models. Maximum temperature, minimum temperature, and dew point temperature data at 4 × 4 km^2^ were obtained from the Parameter-elevation Regressions on Independent Slopes Model (PRISM) Climate Group.^18^ Precipitation data at 4 × 4 km^2^ were obtained from the Gridded Surface Meteorological (gridMET) reanalysis product.^19^ To be consistent with the wildfire-specific PM_2.5_ data, we first extracted values of these meteorological variables at the population-weighted centroids of each ZCTA and then calculated the average across all ZCTAs within San Francisco County.

### Data analysis

We implemented 3 modeling approaches: ARIMA, NNETAR, and Prophet-XGBoost. Our full dataset comprised daily counts of respiratory hospitalizations and associated predictor variables in San Francisco County from January 1, 2009, through December 31, 2018. We divided the dataset into 3 segments: a training period (January 1, 2009, to November 7, 2016), a testing period (November 8, 2016, to November 7, 2018), and a postevent period (during and after the smoke event; November 9, 2018, to December 31, 2018) (Figure 1). All statistical analyses were conducted with R software (version 4.4.0) using the *tidymodels* and *modeltime* packages. The datasets and R scripts used in this study are publicly available at https://github.com/benmarhnia-lab/Two_Stage_ML_ITS.

#### Modeling approaches

##### ARIMA

We first used a traditional ARIMA model to capture the main components of the time series like the trend and seasonality. The ARIMA approach assumes that the future value of a variable is a linear function of several past observations, past forecast errors, and a random error term.^20^ The ARIMA model can be mathematically defined as follows:


$$ {y}_t=c+{\sum_{i=1}^p}{\mathrm{\phi}}_i{y}_{t-i}+{\sum_{j=1}^q}{\mathrm{\theta}}_j{\mathrm{\varepsilon}}_{t-j}+{\mathrm{\varepsilon}}_t $$

where $c$ is a constant, ${\phi}_i$ are the coefficients of the autoregressive terms for $i=1,2,\dots, p$ (the order of the autoregressive part), ${\theta}_j$ are the coefficients of the moving average terms for $j=1,2,\dots, q$ (the order of the moving average part), and ${\varepsilon}_t$ represents the error term related to random noise at time $t$.^20^

The ARIMA model tuning explored both nonseasonal and seasonal components. For nonseasonal parameters, the autoregressive (AR) order and moving average (MA) order were both tuned between 1 and 3, while differencing was tuned between 0 and 2. For seasonal components, both AR and MA orders were tuned between 0 and 3, with seasonal differencing limited to 0 and 1. The tuning process used a space-filling design with 100 combinations. The model also incorporated Fourier terms for yearly seasonality with 3 components to handle periodic patterns.

##### Neural network autoregressive model

Neural networks are flexible frameworks for modeling nonlinear problems.^21^ Specifically, we proposed using a simple feed-forward neural network. This model consists of a network of $n$ processing units (neurons) organized in layers and connected by weighted links. The output of the network can be expressed by the following equation for a single hidden layer network:


$$ {y}_t={\alpha}_0+\sum_{j=1}^s\left({\alpha}_jg\left({\beta}_{0j}+\sum_{i=1}^r{\beta}_{ij}{x}_{t-i}\right)\right)+{\varepsilon}_t $$

Where ${x}_{t-i}$ represents the input at time $t-i$, ${\alpha}_j$ (for $j=0,1,2,\dots, s$) are the output layer weights, and ${\beta}_{ij}$ (for $i=1,2,\dots, r$; $j=1,2,\dots, s$) are the weights of the connections from the input layer to the $j$-th neuron in the hidden layer. The term $r$ denotes the number of input nodes, and $s$ is the number of hidden nodes. The function $g(x)$, often chosen as the logistic sigmoid function, is the activation function for the hidden layer and is defined as:


$$ g(x)=\frac{1}{1+\exp \left(-x\right)} $$

This function introduces nonlinearity into the model, enabling it to capture complex patterns in the data beyond what is possible with linear models. The error term ${\varepsilon}_t$ represents random noise.

The NNETAR tuning focused on both network architecture and time series components. The network architecture parameters included hidden units (8-20 nodes), number of networks (40-100), and penalty term (0.01-0.1) for regularization. For time series aspects, it tuned seasonal AR (1-4) and nonseasonal AR (1-6) components. The training process was configured with 50-200 epochs. The tuning process used a space-filling design with 75 combinations, allowing for efficient exploration of the hyperparameter space. The model also incorporated additional temporal features implemented via time series signatures.

##### Prophet-XGBoost

Finally, we used a hybrid approach combining the Prophet and XGBoost algorithms. According to Zhang,^21^ hybrid methodology suggests that by combining different models, the different underlying patterns of the time series of respiratory hospitalizations ${Y}_t$ can be captured. This approach suggests that every model can be expressed in the following way:


$$ {Y}_t={L}_t+{N}_t $$

where ${L}_t$ denotes the linear component and ${N}_t$ denotes the nonlinear component. This allows great flexibility to create coupled models whose components can be optimally chosen. Thus, initially, from the adjustment of the linear model, we would obtain residuals that capture the nonlinear patterns. Let ${e}_t$ denote the residual at time $t$ from the linear model, then


$$ {e}_t={Y}_t-{\hat{L}}_t $$

A suitable model can be used to analyze these residuals to identify and represent the nonlinear patterns as follows:


$$ {\hat{N}}_t=f\left({e}_{t-j}\right)+{\varepsilon}_t\kern1em for\ j=1,\dots, n $$

where $f$ is a nonlinear function defined by the nonlinear model and ${\varepsilon}_t$ is the random error. Given all the above, the values predicted by the hybrid model would be as follows:


$$ {\hat{Y}}_t={\hat{L}}_t+{\hat{N}}_t $$

For this study, Prophet and XGBoost models were combined under the hybrid methodology framework to improve the prediction performance in the pre-event periods.

Prophet is an additive regression model developed by Facebook for forecasting time series data. It decomposes time series into 3 main components: trend, seasonality, and holidays. The trend component is modeled using a logistic growth curve to accommodate saturated growth patterns, while seasonal effects are captured through Fourier series to model cyclical behavior. The holiday component allows for the incorporation of specific events or occasions that can influence the time series data.^22^ The model can be expressed as:


$$ y(t)=g(t)+s(t)+h(t)+{\varepsilon}_t $$

Where $g(t)$ represents the trend function, $s(t)$ is the seasonal component that captures periodic changes, $h(t)$ accounts for the effects of holidays, and ${\varepsilon}_t$ is the error term.

XGBoost represents an advanced implementation of gradient boosting, where predictive models are built as an ensemble of weak learners, typically decision trees. It operates by consecutively adding predictors to an ensemble, with each new predictor correcting its predecessor’s errors through gradient descent optimization. XGBoost minimizes a regularized objective function that balances prediction error and model complexity, ensuring both accuracy and simplicity in the final model.^23^ The predictive model in XGBoost is formalized as follows:


$$ {\hat{y}}_i={\sum_{k=1}^K{f}_k}\left({x}_i\right),\kern1em {f}_k\in \varPhi $$

where ${\hat{y}}_i$ is the prediction for the $i$-th instance, ${x}_i$ represents the features of the $i$-th instance, $K$ is the number of trees in the model, ${f}_k$ represents the individual decision trees, and $\varPhi$ is the space of all possible regression trees.

The hybrid Prophet-XGBoost model combines these approaches into a robust forecasting framework. Prophet decomposes the time series into trend, seasonality, and holiday components, providing a structured representation of temporal patterns. Then, XGBoost augments this framework by modeling the residuals from the Prophet model through an ensemble of decision trees, minimizing a regularized objective function that balances prediction error and model complexity. The hybrid implementation leverages Prophet’s strength in capturing explicit temporal patterns while using XGBoost’s ability to identify complex relationships in the residuals and additional features.

In our analysis, this hybrid approach was configured with tunable parameters including Prophet’s changepoint_range (0.4-0.8), prior_scale_changepoints (0.01-0.5), and prior_scale_seasonality (0.1-2.0), with fixed linear growth and yearly seasonality. The XGBoost component was configured for regression with gradient boosting (implied by the engine choice), tuning key parameters including number of variables per split (mtry: 4-25), minimum node size (min_n: 1-12), tree depth (8-20), learning rate (0.001-0.1), loss reduction (10^−12^ to 10^2^), and early stopping iterations (10-30). The tuning process used a space-filling design with 100 combinations. The model incorporated additional features through time series signatures and 7-day lags of nonsmoke PM_2.5_ and considered Fourier terms for yearly seasonality with 3 components to handle periodic patterns.

#### Model training and performance assessment

To maintain temporal dependencies, we implement a structured expanding-window time series cross-validation framework on the training data (January 1, 2009 to November 7, 2016). It started with a 2.9-year initial training window followed by a 12-month validation window. We generated 5 sequential folds with nonoverlapping validation window positioned at different times during the training period. The training data in each subsequent fold incorporates all previous training data plus additional 12-month observations (Figure S1).

We evaluate our 3 modeling approaches. Each methodology underwent extensive parameter optimization, exploring 100 parameter configurations for ARIMA, 75 for NNETAR, and 100 for Prophet-XGBoost. The optimal parameter set for each methodology is determined by minimizing the Root Mean Square Error (RMSE) across the cross-validation folds.

Then, for each modeling approach, we evaluated each optimized model using the testing data (November 8, 2016 to November 7, 2018). Model performance was comprehensively evaluated based on coefficient of determination (*R*^2^), mean absolute error (MAE), RMSE, mean absolute percentage error (MAPE), and symmetric mean absolute percentage error (SMAPE).

#### Excess hospitalization calculation and uncertainty measurement

The best performing modeling approach in the testing period was then used to predict respiratory hospitalizations for the whole study period. To quantify prediction uncertainty, we employed a Moving Block Bootstrap (MBB) approach,^24^^,^^25^ performing 1000 model iterations. In brief, the observed series of daily hospitalizations during the pre-event period were decomposed into predicted values by the model and the residuals. The residuals of pre-event data were divided into $n-L+1$ overlapping blocks, where $n$ is the length of the pre-event data (3958 days) and $L$ is the size of each block. In each iteration, we sampled $n/L$ blocks with replacement and concatenated them to form a bootstrapped residual series. These bootstrapped residuals were added back to the model predictions to yield a new pseudo-time series to which the pretrained model was reapplied to generate predictions after the event occurrence. The 95% empirical CIs (eCIs) were derived from the empirical distribution of these 1000 predictions, taking the 2.5th and 97.5th percentiles as the lower and upper bounds, respectively. Note that this approach does not enforce symmetry around the original point prediction, so the central estimate may not lie exactly at the midpoint of the intervals, but its simplicity and minimal assumptions have made it a common choice in time series applications.^26^^-^^28^ We selected $L=14$ based on a nonparametric plug-in rule proposed by Lahiri et al.^29^ This choice was further confirmed when we ran the MBB procedure for a range of block size (1-100), inspected how the width of the 95% CIs change, and chose the smallest $L$ that yields stable interval widths (Figure S2). By sampling blocks of contiguous observations rather than individual observations, the MBB approach allows the bootstrap samples to retain the correlation structure within blocks, making it more suitable for time series data.^24^^,^^25^

The predicted hospitalizations during and after the smoke event represents the expected hospitalization counts under the counterfactual scenario in which the smoke event did not occur. The excess hospitalizations resulting from the smoke event were then calculated by the difference between the observed and expected hospitalizations. The 95% eCIs of the estimated excess hospitalizations were calculated based on the 95% eCIs of the model predictions. We calculated both daily excess hospitalizations and the total excess hospitalizations during the whole period of the smoke event.

## Results

During the 2018 wildfire smoke event (November 9-20, 2018), a total of 738 respiratory hospitalizations were recorded in San Francisco County, CA. The average daily total PM_2.5_ concentration was 75.6 μg/m^3^, of which approximately 88% was from wildfire smoke.

Table 1 presents the performance metrics for the 3 models evaluated on both training and testing datasets. In the training dataset, the NNETAR model showed an overall top performance, with an *R*^2^ of 0.84 and consistently the lowest error measures, while the ARIMA model showed the poorest performance. In the testing dataset, Prophet-XGBoost showed the best performance, with the highest *R*^2^ (0.69) and the lowest MAE, RMSE, and SMAPE. Overall, although both ARIMA and NNETAR models showed reasonable performance, they were outperformed by the Prophet-XGBoost model in the testing dataset. Therefore, Prophet-XGBoost was selected to predict the counterfactual scenario in the postevent period.

**Table 1:** Performance metrics of the ARIMA, NNETAR, and Prophet-XGBoost model.

	**Training (January 1, 2009 to November 7, 2016)**			**Testing (November 8, 2016 to November 7, 2018)**		
	**ARIMA**	**NNETAR**	**Prophet-XGBoost**	**ARIMA**	**NNETAR**	**Prophet-XGBoost**
*R* ^2^	0.71	0.84	0.76	0.65	0.58	0.69
MAE	7.08	5.36	6.49	8.65	9.44	8.64
RMSE	9.19	6.88	8.39	11.82	13.03	11.15
MAPE	0.14	0.11	0.13	0.16	0.16	0.17
SMAPE	0.13	0.10	0.12	0.16	0.17	0.16

Abbreviations: MAE, mean absolute error; MAPE, mean absolute percentage error; *R*^2^, coefficient of determination; RMSE, root mean square error; SMAPE, symmetric mean absolute percentage error.

**Figure 3:**
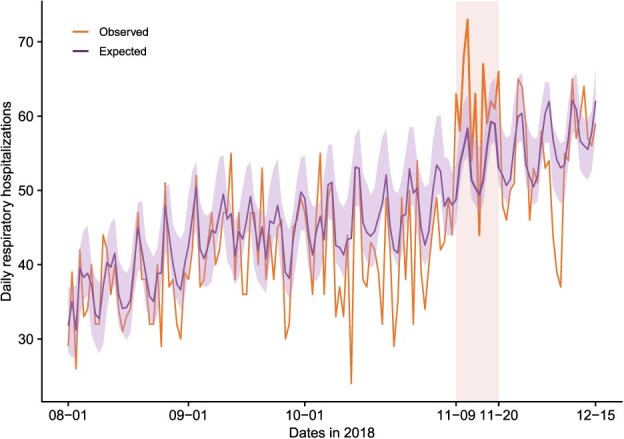
Daily observed and expected respiratory hospitalizations in San Francisco County, before, during, and after the 2018 wildfire smoke event. This figure illustrates the time series of the daily observed (orange line) and expected (the counterfactual; purple line) respiratory hospitalizations in San Francisco County, before, during, and after the 2018 wildfire smoke event. The expected hospitalizations were predicted using the Prophet-XGBoost model. The shaded rectangle marks the period of the smoke event (November 9-20, 2018). The shaded area around the expected hospitalizations represents the 95% empirical CI for the expected hospitalizations.

The hybrid Prophet-XGBoost model predicted a total of 646 (95% eCI, 613-714) expected respiratory hospitalizations in San Francisco County from November 9 to 20, 2018, under the counterfactual scenario in which the smoke event did not occur. Therefore, by comparing the observed and expected hospitalizations, we estimated a total of 92 (95% eCI, 24-125) excess respiratory hospitalizations resulting from the 2018 wildfire smoke event in San Francisco County (approximately 8 excess hospitalizations per day), accounting for approximately 12.5% of the total observed hospitalization count during the event period (Figure 3; Table 2). The estimated daily excess respiratory hospitalizations during the 2018 wildfire smoke event were also listed in Table 2. The highest attributable fraction of respiratory hospitalizations occurred on November 16 (approximately 23.5%), followed by the first day of the smoke event (November 9; approximately 22.7%) and November 12 (approximately 20.1%).

**Table 2:** Estimated excess respiratory hospitalizations during the 2018 wildfire smoke event in San Francisco County.

** Date/period**	**Observed hospitalizations (count)**	**Expected hospitalizations (count, 95% eCI)**	**Excess hospitalizations (count, 95% eCI)**	**Attributable fraction^a^ (%, 95% eCI)**
**Daily estimates during the event**				
November 9, 2018	63	49 (46-53)	14 (10-17)	22.7 (15.2-27.7)
November 10, 2018	58	54 (51-59)	4 (−1 to 7)	7.6 (−1.0 to 12.7)
November 11, 2018	68	56 (54-62)	12 (6-14)	17.9 (9.0-20.8)
November 12, 2018	73	58 (54-63)	15 (10-19)	20.1 (13.9-25.4)
November 13, 2018	54	51 (49-57)	3 (−3 to 5)	4.8 (−6.2 to 9.4)
November 14, 2018	63	50 (48-57)	13 (6-15)	20.1 (10.2-23.9)
November 15, 2018	44	49 (47-56)	–5 (−12 to −3)	−12.3 (−27.0 to −6.8)
November 16, 2018	67	51 (48-57)	16 (10-19)	23.5 (15.5-28.3)
November 17, 2018	59	56 (53-61)	3 (−2 to 6)	5.4 (−4.1 to 10.6)
November 18, 2018	62	59 (56-64)	3 (−2 to 6)	4.4 (−3.9 to 9.6)
November 19, 2018	61	59 (57-65)	2 (−4 to 4)	3.3 (−6.9 to 7.3)
November 20, 2018	66	53 (51-60)	13 (6-15)	19.7 (9.2-22.8)
**Total estimates during the event**				
November 9-20, 2018	738	646 (613-714)	92 (24-125)	12.5 (3.2-17.0)

The expected hospitalizations were predicted using the Prophet-XGBoost model.

^a^ Abbreviations: Attributable fraction, fraction of observed hospitalizations attributable to the smoke event; eCI, empirical CI.

In a secondary analysis, we included November 8, 2018 (the day the Camp Fire began in its source location) as the beginning of the wildfire smoke event period. The estimated excess respiratory hospitalizations during this extended period were 88 (95% eCI, 15-125), which is similar to the estimate obtained using the primary definition of the smoke event period. In the week following the smoke event (November 21-27, 2018), the estimated difference between the total observed and expected hospitalizations was null (−1 [95% eCI, −42 to 19]).

## Discussion

In this paper, we proposed a hybrid approach combining ML and a 2-stage ITS analysis. We applied this methodology to assess the health impacts of the 2018 wildfire smoke event in San Francisco County, CA, serving as a case study. Using the hybrid Prophet-XGBoost model to generate the hospitalization trend under counterfactual scenario, we estimated that this 12-day smoke event resulted in a total of 92 (95% eCI, 24-125) excess respiratory hospitalizations in this county, which accounted for about 12.5% of the observed hospitalizations. This innovative method augments traditional ITS analysis by integrating ML algorithms to generate robust counterfactual trends, allowing for more accurate causal inference of the impacts of extreme weather events. Additionally, using ML models to generate counterfactuals allows for more flexible assumptions as compared to the stringent assumptions of linearity and seasonality under the traditional ITS framework.

Our findings on the effects of wildfire smoke events on respiratory health are overall consistent with the broader literature.^14^^,^^15^ Specifically, the magnitude of the effect estimate in our study aligns with previous research. For example, a previous study investigating the health effects of the same 2018 wildfire event in California using a synthetic control approach reported approximately 98 increased respiratory hospitalizations in San Francisco County during the event.^30^ Another study utilized a 2-stage ITS design to estimate the healthcare utilization effects of the December 2017 Lilac Fire in San Diego County among pediatric patients, and found that the Lilac Fire was associated with approximately 16 excess respiratory visits per day (underlying population size: approximately 690 000 children aged 0-19),^9^ which is higher than our estimate of about 8 excess hospitalizations per day (underlying population size: about 800 000 people of all age groups). However, this direct comparison of estimated excess hospital visits across studies focusing on different smoke events should be interpreted with caution due to multiple factors, such as the difference in wildfire characteristics, potential heterogeneity in wildfire effects across age groups, whether the region was directly burned or only exposed to smoke, and possible differences in the chemical composition of the smoke. The overall consistency between results from existing methods and the newly proposed hybrid approach provides robust support for the acute respiratory impacts of wildfire smoke. Our hybrid methodology, which combines ML and a 2-stage ITS analysis, can be utilized alongside other causal inference designs to generate more robust estimates of the impacts of extreme weather events or the effectiveness of interventions.

Our study presents a novel application of ML techniques within an ITS framework to evaluate the health impacts of extreme weather events. We showcase the 2-stage hybrid approach and demonstrate its flexibility and robustness of causal inference. This method can be widely applied to other contexts, such as evaluating the effectiveness of policies and interventions. The 2-stage approach can also be easily adapted to staggered interventions^31^ (when no control groups are available), which is a very active area of research in the context of Difference-in-Differences (DID) methods. It can be extended to assess heterogeneity in effect estimates across time or treated units (if multiple treated units receive the treatment in a sequential way).^32^ We also believe this approach can be readily applied to evaluate interventions across diverse health issues and geographical contexts, highlighting its potential as an important tool in public health research.

While our approach marks a significant methodological advancement, it diverges from other analytical methods, such as DID or synthetic control methods. One caveat in applying this approach is the potential overfitting of the ML models. Although overfitting was not an issue in our case study, it may arise in other contexts. However, since the primary purpose of training the ML model is to generate a counterfactual scenario, the potential close alignment of pre-event trends with actual data is of less concern. In addition, unlike standard single-stage ITS models, although the nonparametric and complex modeling strategies in our proposed ML-integrated 2-stage ITS models make them more flexible to unexpected patterns in the data, it makes it more difficult for researchers to explicitly state and test their assumptions about the impact model and the temporal patterns of effects in the postevent period. Further work to address this limitation is warranted in future studies.

## Conclusion

There is an urgent need for more robust and flexible methods to evaluate the impacts of extreme weather events, particularly as climate change exacerbates a range of existing and emerging public health threats. Our approach, which integrates ML techniques with traditional quasi-experimental methods, provides researchers with an enhanced tool for generating counterfactual scenarios. This methodological framework enhances our capacity to assess the impact of events or interventions in real-world situations where obtaining a direct counterfactual may not be possible. The approach presented in this paper offers an innovative method for assessing the effects of extreme weather events and public health interventions, which can provide robust scientific evidence to better inform public health strategies amid evolving environmental and epidemiological landscapes.

## Supplementary material

Supplementary material is available at the *American Journal of Epidemiology* online.

## Supplementary Material

Web_Material_kwaf147

## Data Availability

The datasets and R scripts used in this study are publicly available at https://github.com/benmarhnia-lab/Two_Stage_ML_ITS.
