# Supplementary material for: Two-stage interrupted time series analysis with machine learning: evaluating the health effects of the 2018 wildfire smoke event in San Francisco County as a case study
Source: Am J Epidemiol. 2025 Jul 11;194(10):2936–44. doi: 10.1093/aje/kwaf147 (PMC12527249; doi:10.1093/aje/kwaf147)
Supplement: Web_Material_kwaf147 [file web_material_kwaf147.docx]

**Supplementary Data**

**Two-Stage Interrupted Time Series Analysis with Machine Learning: Evaluating the Health Effects of the 2018 Wildfire Smoke Event in San Francisco County as a Case Study**

Arnab K. Dey, Yiqun Ma, Gabriel Carrasco-Escobar , Changwoo Han, François Rerolle, Tarik Benmarhnia

List of included materials

[Appendix S1: International Classification of Diseases (ICD)-9 and ICD-10 codes to identify respiratory hospitalizations 2](#_Toc202448467)

[Figure S1. Training and validation windows in the cross-validation. 3](#_Toc202448468)

[Figure S2. Changes in widths of 95% empirical confidence intervals with different block sizes in moving block bootstrap. 4](#_Toc202448469)

# Appendix S1: International Classification of Diseases (ICD)-9 and ICD-10 codes to identify respiratory hospitalizations

The ICD codes included to identify a respiratory hospitalization are:

- **ICD-9 codes**: "460", "461", "462", "463", "464", "465", "466", "470", "471", "472", "473", "474", "475", "476", "477", "478", "480", "481", "482", "483", "484", "485", "486", "487", "490", "491", "492", "493", "494", "495", "496", "500", "501", "502", "503", "504", "505", "506", "507", "508", "510", "511", "512", "513", "514", "515", "516", "517", "518", "519".
- **ICD-10 codes**: "J00", "J01", "J02", "J03", "J04", "J05", "J06", "J09", "J10", "J11", "J12", "J13", "J14", "J15", "J16", "J17", "J18", "J20", "J21", "J22", "J30", "J31", "J32", "J33", "J34", "J35", "J36", "J37", "J38", "J39", "J40", "J41", "J42", "J43", "J44", "J45", "J46", "J47", "J60", "J61", "J62", "J63", "J64" ,"J65", "J66", "J67", "J68", "J69", "J70", "J80", "J81", "J82", "J83", "J84", "J85", "J86", "J90", "J91", "J92", "J93", "J94", "J95", "J96", "J97", "J98", "J99"


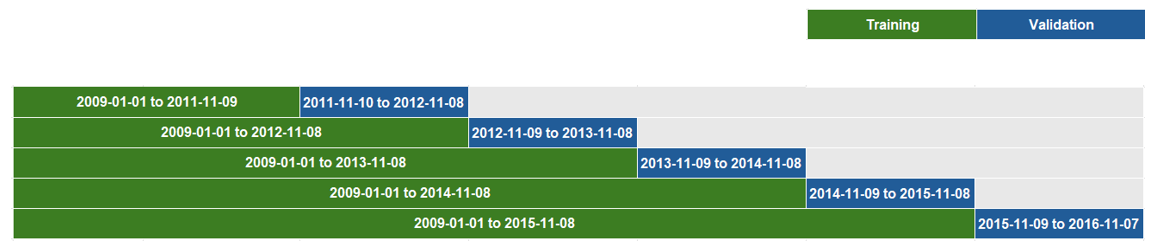


# Figure S1. Training and validation windows in the cross-validation.

This figure illustrates our expanding-window time-series cross-validation framework applied to the training data (January 1, 2009 to November 7, 2016). We generated five slices, each represented by a row in the figure. In the first slice, the training period is approximately 2.9 years (January 1, 2009 to November 9, 2011), followed by a 12-month validation period (November 10, 2011 to November 8, 2012). In each subsequent slice, the previous validation period is added into the training set, and the following 12-month period becomes the new validation window.


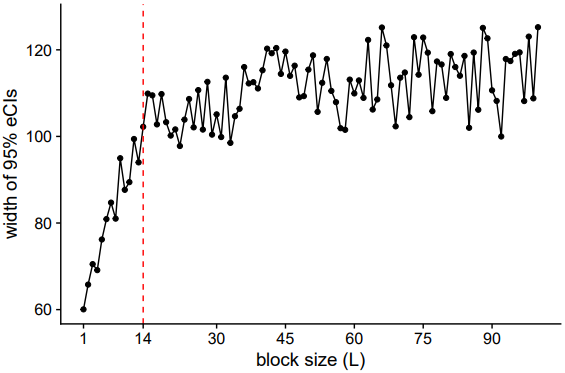


# Figure S2. Changes in widths of 95% empirical confidence intervals with different block sizes in moving block bootstrap.

This figure illustrates how the 95% empirical confidence intervals (eCIs) of predicted total hospitalizations during the smoke event changes as the block size in the moving block bootstrap (MBB) increases. The x axis of this plot is the block size (L) used in the MBB. The y axis is the width of the 95% eCIs (upper - lower). The red dashed line marked the chosen block size (L = 14).
